# Tough metal-ceramic composites with multifunctional nacre-like architecture

**DOI:** 10.1038/s41598-021-81068-z

**Published:** 2021-01-15

**Authors:** Erik Poloni, Florian Bouville, Christopher H. Dreimol, Tobias P. Niebel, Thomas Weber, Andrea R. Biedermann, Ann M. Hirt, André R. Studart

**Affiliations:** 1 Complex Materials, Department of Materials, ETH Zürich, 8093 Zurich, Switzerland; 2 X-Ray Service Platform, Department of Materials, ETH Zürich, 8093 Zurich, Switzerland; 3 Institute of Geological Sciences, University of Bern, 3012 Bern, Switzerland; 4 Institute of Geophysics, ETH Zürich, 8092 Zurich, Switzerland; 5 Present Address: Centre for Advanced Structural Ceramics, Department of Materials, Imperial College London, South Kensington Campus, London, SW7 2AZ UK

**Keywords:** Ceramics, Composites, Mechanical properties

## Abstract

The brick-and-mortar architecture of biological nacre has inspired the development of synthetic composites with enhanced fracture toughness and multiple functionalities. While the use of metals as the “mortar” phase is an attractive option to maximize fracture toughness of bulk composites, non-mechanical functionalities potentially enabled by the presence of a metal in the structure remain relatively limited and unexplored. Using iron as the mortar phase, we develop and investigate nacre-like composites with high fracture toughness and stiffness combined with unique magnetic, electrical and thermal functionalities. Such metal-ceramic composites are prepared through the sol–gel deposition of iron-based coatings on alumina platelets and the magnetically-driven assembly of the pre-coated platelets into nacre-like architectures, followed by pressure-assisted densification at 1450 °C. With the help of state-of-the-art characterization techniques, we show that this processing route leads to lightweight inorganic structures that display outstanding fracture resistance, show noticeable magnetization and are amenable to fast induction heating. Materials with this set of properties might find use in transport, aerospace and robotic applications that require weight minimization combined with magnetic, electrical or thermal functionalities.

## Introduction

Artificial materials that mimic the nacreous architecture of mollusk shells are prominent examples of how evolved hierarchical structures made by living organisms can be harnessed to fabricate synthetic counterparts with outstanding properties and new functionalities^1–3^. By combining design principles of nacre’s brick-and-mortar structure with the rich variety of chemistries available in synthetic systems, current nacre-like materials exhibit mechanical properties that even surpass those of the natural counterparts^4–9^. As opposed to conventional materials, such bio-inspired structures can be designed to showcase antagonistic properties, such as high stiffness and fracture toughness, that are not accessible through the optimization of chemical compositions alone^7,10,11^. Despite these promising prospects, a broader application of such bio-inspired materials requires the development of manufacturing technologies that ensure up-scalability and robust structural control.

Several processing technologies have been developed in the last decade to manufacture synthetic films and bulk materials with nacre-like brick-and-mortar architecture^11,12^. Some of the several approaches proposed include the magnetic or shear-induced alignment of pre-formed platelets into layered structures^5,6,13,14^, the sequential deposition of individual layers into stacked composites^15–18^ and the freeze-casting of suspensions into lamellar architectures^3,19–21^. The resulting anisotropic structures can be consolidated by sintering and/or infiltrated with a matrix to generate the desired brick-and-mortar architecture. The use of platelets has become particularly attractive due to the very fine microstructures obtained after consolidation and the increasing availability of two-dimensional materials with different chemical compositions^5,6,18,22^.

Recent research efforts have been dedicated to increase further the fracture toughness and stiffness of nacre-like bulk materials or to complement these mechanical properties with additional functionalities. By utilizing building blocks with matched optical properties, nacre-like composites combining for instance crack growth resistance with optical transparency have been reported^22^. In another example, nacre-inspired materials featuring a graphene percolating network as continuous phase have been developed to imbue brick-and-mortar structures with self-sensing capabilities^23^. In addition to these functionalities, studies have also been conducted to enhance the mechanical properties of nacreous materials by utilizing stiffer and tougher metals as the mortar phase between stiff ceramic bricks^7,20,24–30^. Because of the plastic deformation of the metallic phase during fracture, composites containing nickel, aluminum or amorphous metal alloys between alumina platelets have been shown to feature remarkable mechanical stiffness and enhanced resistance to crack growth^7,20,31^. The use of metals as continuous phase also opens the opportunity to incorporate magnetic, electrical and thermal functionalities that have not yet been fully explored in nacre-like composites with mechanically-robust bulk geometries.

Here, we report a processing route to create metal-coated oxide platelets that can be assembled into bulk nacre-like composites that combine enhanced fracture toughness with magnetically-responsive functionalities. This is achieved by utilizing metallic iron as the continuous phase between alumina platelets organized in a brick-and-mortar architecture. The processing route used to create this architecture and the properties of the resulting multifunctional composites are thoroughly investigated in this work. First, we describe a sol–gel route and a thermal reduction protocol to controllably coat pre-existing alumina platelets with well-defined fractions of iron. Next, the mechanical properties of nacre-like composites assembled from the pre-coated platelets are systematically assessed using state-of-the-art mechanical testing procedures. Finally, the magnetic properties of the composites are characterized and explored to create exemplary light-weight materials that can be inductively heated in an oscillating magnetic field.

## Results and discussion

**Figure 1:**
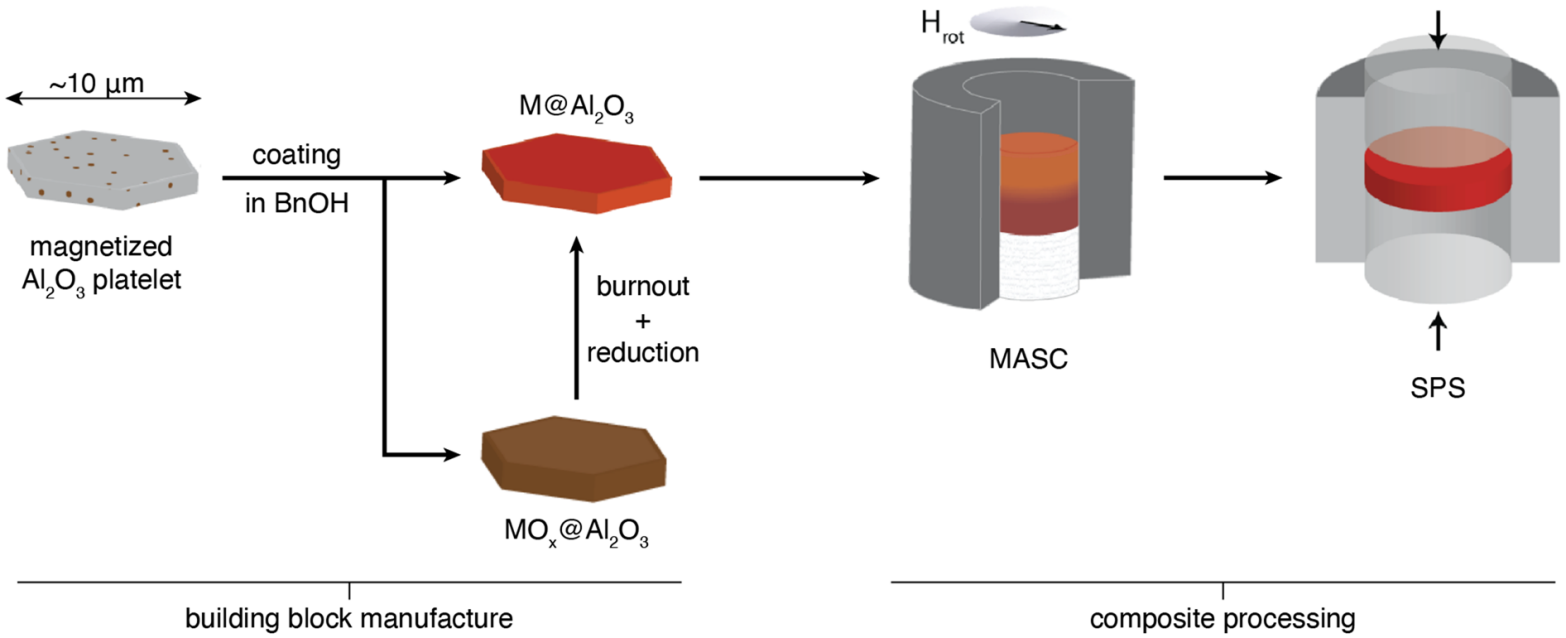
Processing route used for the fabrication of metal–ceramic nacre-like composites through the assembly of coated platelets followed by densification at high temperature. BnOH: benzyl alcohol. M: metal. MO_x_: metal oxide. H_rot_: rotating magnetic field. MASC: magnetically-assisted slip casting. SPS: Spark Plasma Sintering.

The formation of metal-coated platelets and their assembly into nacre-like metal-ceramic composites is achieved through a processing route that includes: (i) coating of platelets with a metallic or an oxide layer, (ii) possible reduction of the oxide layer to generate metal-coated platelets, (iii) assembly of the metal-coated platelets into nacre-like architectures, and (iv) pressure-assisted sintering of the nacre-like structure into tough multifunctional composites (Fig. 1).

**Figure 2:**
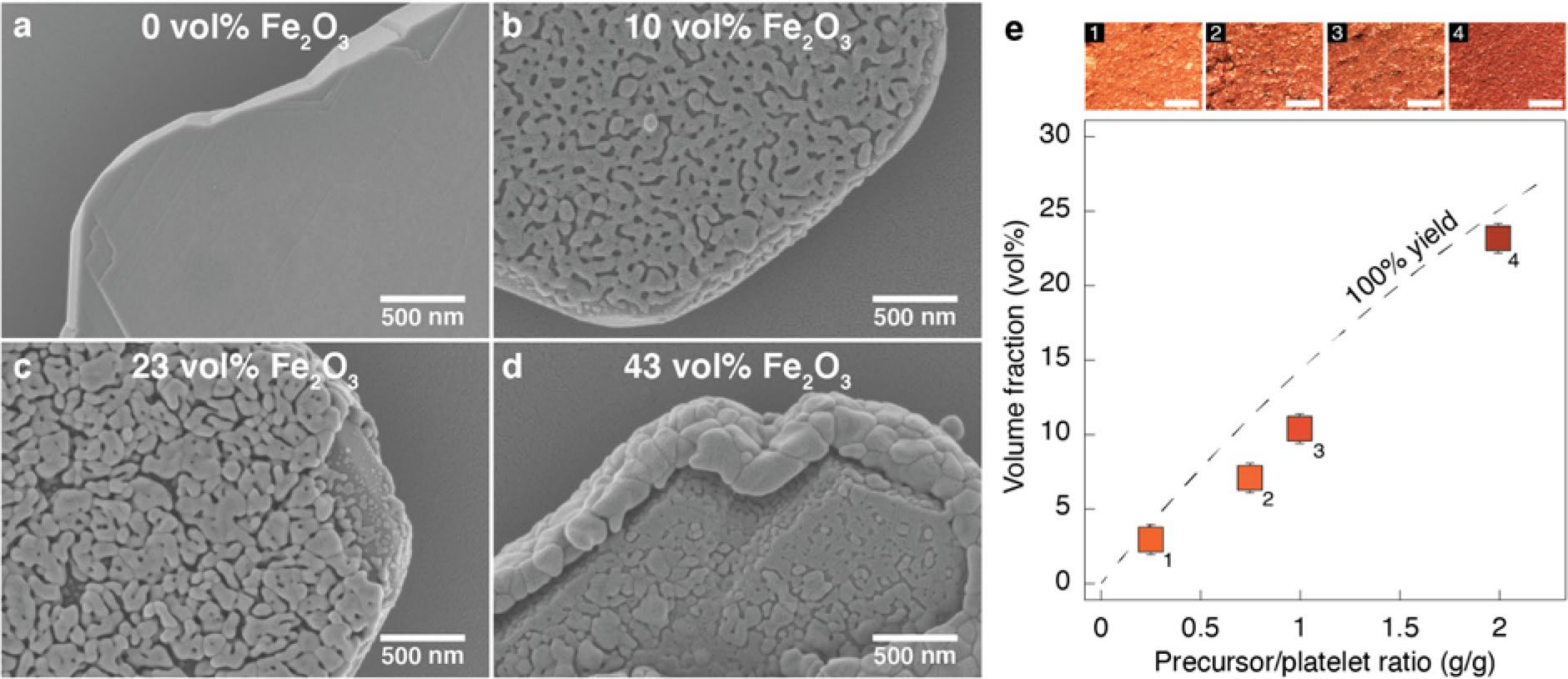
Alumina platelets coated with different concentration of hematite. Scanning electron micrographs of (**a**) bare platelets and of platelets coated with (**b**) 10, (**c**) 23 and (**d**) 43 vol% of hematite. The imaged platelets were obtained after the sol–gel coating procedure followed by a burn-out step. Platelets shown in (**b**) and (**c**) were prepared using precursor/platelet mass ratios of 1 and 2, respectively. The high fraction of hematite formed on the platelet displayed in (**d**) results from two successive coating reactions performed with the same powder. (**e**) Volume fraction of hematite formed on the platelet surface as a function of the initial precursor/platelet mass ratio. The dotted line indicates the predicted volume fraction if all the iron added as precursor for the sol–gel reaction is converted into oxide particles on the surface of the platelets. Optical images of the powders synthesized with increasing precursor/platelet ratios. Scale bars: 500 μm.

Coating of the platelets with an oxide layer is accomplished through a non-aqueous sol–gel reaction using a metal–organic precursor dissolved in benzyl alcohol^32,33^. Taking iron acetylacetonate as precursor, a thin homogeneous layer of magnetite (Fe_3_O_4_) nanoparticles is formed on the surface of the alumina platelets during the sol–gel reaction at 180°C^34^. The reaction is followed by removal of the solvent and burn-out of the organic phase in air at 700 °C (Fig. 2). X-Ray diffraction of the coated platelets reveals that the magnetite coating is converted to crystalline hematite (Fe_2_O_3_) during the burnout process^35^. The bare alumina platelets present flat surfaces (Fig. 2a), while the hematite forms a uniform percolating network comprised of nanoparticles on the surface of the platelets (Fig. 2b,c). Bonds between the nanoparticles are likely caused by partial sintering during the burn-out step.

Importantly, the volume fraction of hematite particles on the platelet surface can be controlled by tuning the relative amount of precursor used in the sol–gel reaction (Fig. 2e). By comparing the volume fraction of hematite measured on the surface with values predicted assuming a 100% yield, we conclude that not all the iron oxide nanoparticles formed during the reaction are eventually adsorbed on the surface. This is further confirmed by the deep black color of the solvent obtained after filtration of the platelets at the end of the coating procedure. Despite the incomplete adsorption, the fraction of iron oxide nanoparticles formed on the surface is not far from the theoretical predictions for 100% yield (Fig. 2e). This allows us to use the theoretical prediction as a good first estimate of the precursor/platelet ratio needed to reach a given volume fraction of hematite on the platelet surface. In order to achieve hematite concentrations higher than 25%, successive sol–gel reactions can be performed on the same powder. This successive coating yields additional layers of hematite on the top of already-coated platelets (Fig. 2d) and increases the coating volume fraction up to 43% while minimizing the fraction of nanoparticles formed in solution. The increase in concentration of iron oxide formed on the surface of the platelets prepared with higher precursor contents is readily visible from a change in the color of the filtrated and dried powder from light to dark red (Fig. 2e).

**Figure 3:**
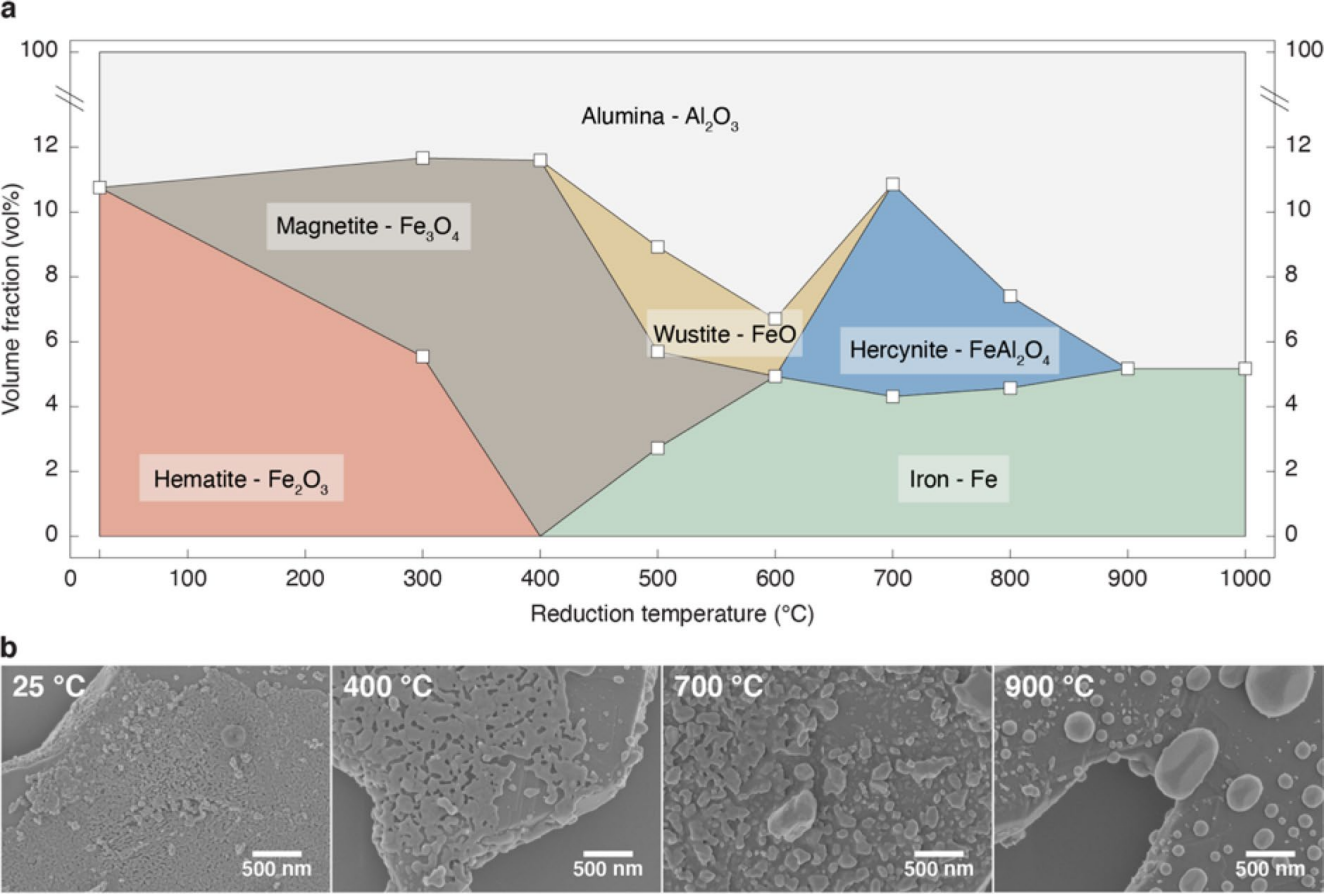
Crystallography and morphology of the platelet coating after heat treatment at distinct temperatures under reducing atmosphere in a small-batch process. (**a**) Volume fraction of each crystalline phase present in coated platelets subjected to different reduction temperatures for 8 h. The initial coated platelets consist of 89% Al_2_O_3_ and 11% Fe_2_O_3_. The lines between experimental data points are guides to the eye. (**b**) SEM images of the platelet coating at selected temperatures, highlighting the morphological changes associated with the phase transformations of the initial hematite particles. Magnification: 85 k ×.

The alumina platelets coated with hematite nanoparticles were further processed in order to transform the oxide layer into a metallic coating. This was performed by heat treating the oxide-coated platelets in a reducing 5% H_2_/N_2_ atmosphere at high temperatures. To identify the different phases formed during the reduction process and establish the minimum temperature required to form the metallic iron coating, we conducted *ex-situ* X-Ray diffraction analysis on platelets coated with 10 vol% hematite and treated for 8 h at distinct temperatures up to 1000 °C. The experiments show that five different crystalline phases may be present in the coating depending on the temperature used for reduction of the initial hematite particles. Rietveld refinement of the obtained diffractograms allows us to quantify the amount of the various phases formed after the reduction step (Fig. 3).

Our quantitative analysis shows that the initial hematite phase (Fe_2_O_3_) is partially and fully reduced to magnetite (Fe_3_O_4_) after reduction at 300 and 400 °C, respectively. Further reduction of the magnetite phase into either wustite (FeO) or metallic iron is then observed when the platelets are thermally treated between 400 and 600 °C. The volume fraction of metallic iron in the coating after reduction at 600 °C is estimated to be 4.7 vol%. Thermal treatment at the higher temperature of 700 °C leads to the formation of the alumina-iron spinel FeAl_2_O_4_. This mixed oxide phase, called hercynite, is likely formed by the diffusion of iron atoms to the alumina crystalline phase at the platelet/coating interface. When the temperature is increased beyond 700 °C, the fraction of the spinel phase decreases until it is fully converted into metallic Fe at 900 °C. The iron content of 5.2 vol% measured by X-Ray diffraction after full reduction above 900 °C is in good agreement with the nominal value of 5.4 vol% estimated from the initial fraction of hematite present in the platelet coating. This indicates that our reduction protocol provides an effective means to create metal-coated platelets starting from sol–gel precursors. Because the non-aqueous sol–gel route utilized to coat the platelets is applicable to a wide range of chemistries, it can also be applied to tailor the composition of the coating. Indeed, platelets coated with copper, copper oxide, tungsten oxide, molybdenum, molybdenum oxide or nickel could be successfully prepared by simply changing the metal precursor in the initial sol–gel mixture (Figure S1).

In addition to changes in the crystalline phases, the reduction process was also found to affect the morphology of the platelet coating. We monitored these morphological transformations by examining scanning electron microscopy (SEM) images of platelets treated at different temperatures in a small-batch process (Fig. 3b). The images reveal that the thermal treatment under reducing atmosphere promotes sintering between the initial particles, thus increasing the characteristic size of the grains in the platelet coating. As expected, such coarsening effect is more pronounced at higher temperatures. Importantly, grains were found to wet the surface of the alumina platelets if the composition of the coating contained hercynite or any of the iron oxides. By contrast, the formation of a fully metallic coating at 900 °C led to de-wetting and the formation of large iron particles on the platelet surface.

X-Ray diffraction and SEM imaging of platelets containing higher fractions of hematite showed similar trends in terms of crystalline phases and coating morphology. However, we noticed that alumina platelets coated with the highest hematite fraction of 43 vol% completely lose their morphology and high aspect ratio when reduced at a temperature of 700 °C or higher (Figure S2). Although further investigation is needed to elucidate this effect, the observed morphological change is probably related to the full transformation of the alumina into the hercynite phase. Guided by these experimental results, we selected a reduction temperature of 1000 °C for the preparation of iron-coated platelets with initial hematite fractions of 10 and 23 vol% (Fig. 2b,c). To preserve the platelet morphology while maximizing the fraction of metallic phase in the coating, platelets containing an initial hematite content of 43 vol% (Fig. 2d) were reduced at the temperature of 600 °C. The wustite phase observed in platelets reduced at this lower temperature was found to eventually transform into hercynite or iron at the high sintering temperature of 1450 °C used later for the consolidation of the nacre-like composites. The knowledge gained in this small-batch study was eventually used to establish an up-scaled process for the preparation of the large quantities of coated platelets necessary for the metal-ceramic composites (see “Methods” section).

**Figure 4:**
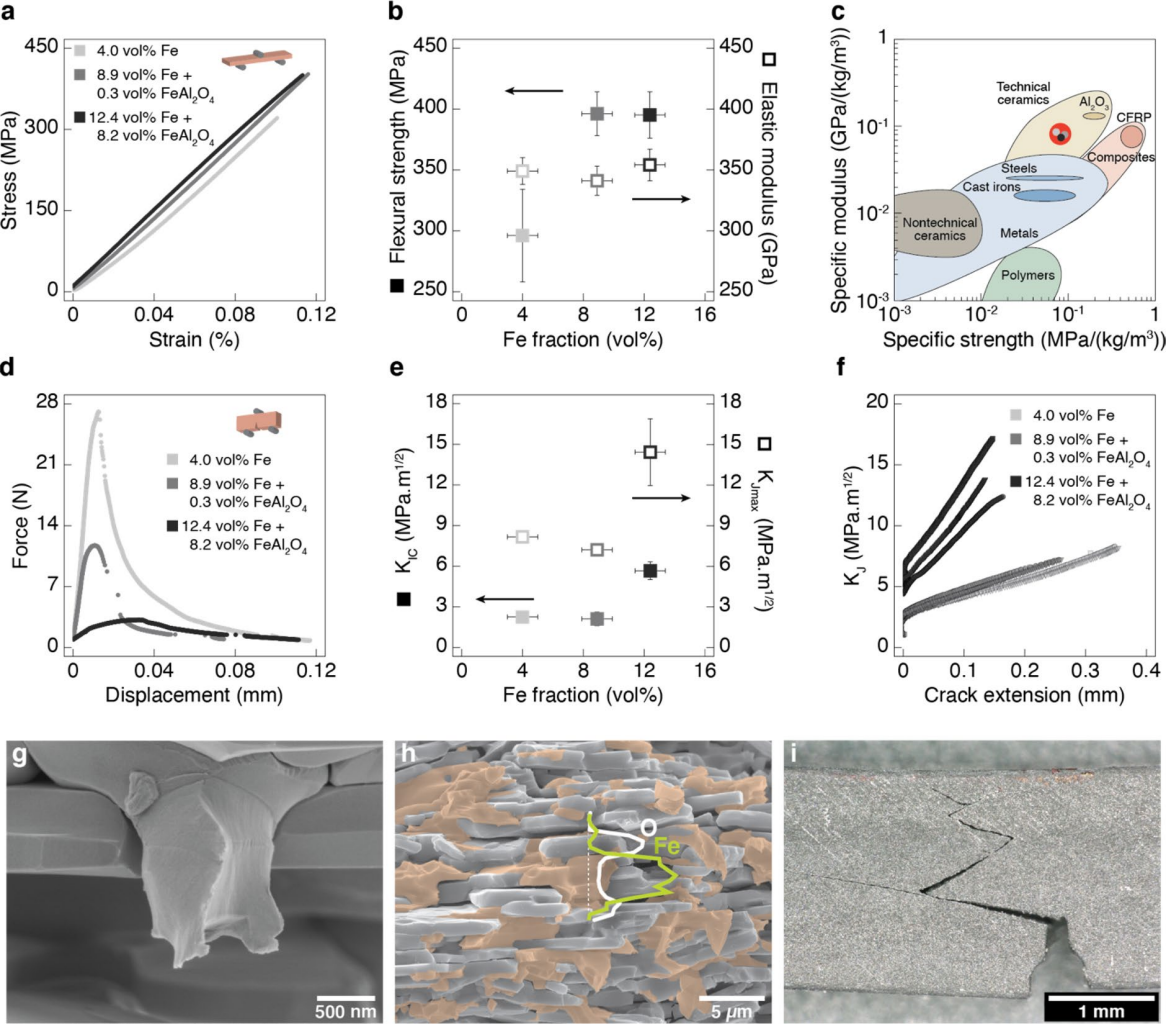
Mechanical properties of metal-ceramic composites with nacre-like architecture. (**a**) Stress–strain curves obtained from three-point bending tests on un-notched specimens containing different iron fractions. (**b**) Effect of the metal fraction on the flexural strength and the elastic modulus of nacre-like composites. (**c**) Ashby plot depicting the specific elastic modulus and specific strength of our metal-ceramic composites in comparison to literature data for conventional materials. (**d**) Force–displacement data obtained for single-edge notched beam samples with varying iron content. (**f**) Effect of the metal fraction on the critical stress intensity factor for crack initiation (*K*_*IC*_) and the maximum intensity factor after crack propagation (*K*_*Jmax*_). (**e**) Stress intensity factor (*K*_*J*_) of the composite as a function of the propagated crack length (R-curve). (**g**,**h**) Scanning electron microscopy (SEM) images of the fracture surface of a specimen containing 12.4 vol% Fe highlight (**g**) the plastic deformation of the metallic phase and (**h**) the homogeneous distribution of metallic iron within the structure. Magnification: 88 k ×. In image (**h**) the EDX intensity spectra for Fe and O elements across a selected line is displayed on top of a false coloured micrograph to illustrate the distribution of metallic and ceramic phases in the structure. Magnification: 10 k ×. (**i**) Light microscopy image of the fracture generated in a notched specimen after testing. Magnification: 100 ×.

Our ability to reduce the initial oxide layer of the coated platelets into metallic iron opens the possibility to fabricate nacre-like metal-ceramic composites by assembling the platelets into brick-and-mortar architectures. Because of the magnetic nature of the iron coating, brick-and-mortar architectures were readily obtained using the magnetically-assisted slip casting (MASC) approach^6^. In this process, platelets dispersed in a suspension are aligned with the help of an external magnetic field and collected at the walls of a porous mould to generate structures with highly oriented architectures after casting and drying (Fig. 1). Sintering of such structures under pressure at high temperatures allows for densification of the aligned architecture while maintaining a fine-grained microstructure. Following this procedure, we created metal-ceramic composites with a nacre-like architecture consisting of alumina “bricks” and metallic iron as the “mortar” phase (Fig. 4h). X-Ray diffraction of specimens after sintering confirmed the presence of α-iron in fractions ranging from 4.0 to 12.4 vol% as the main constituent of the mortar phase. In addition to iron, compositions made from platelets originally coated with 23 and 43 vol% of hematite were also found to contain, respectively, 0.3 and 8.2 vol% of hercynite after the sintering process.

Nacre-like composites containing metallic iron as the main mortar phase exhibit high fracture toughness combined with high mechanical strength and elastic modulus. This unusual set of antagonistic properties were assessed by testing notched and un-notched prismatic specimens under three-point bending following standard mechanical characterization protocols^36–38^. Results obtained from the characterization of un-notched samples show that the composites display strong linear elastic response with elastic modulus ranging from 341 to 355 GPa and fracture strength lying between 300 and 400 MPa (Fig. 4a,b). Except for the relatively low strength of the composition with 4.0 vol% Fe, the fraction of the metallic phase was found to have no significant effect on the elastic modulus and fracture strength. Given that the elastic modulus of iron (200 GPa) and alumina (370 GPa) are relatively close, this experimental result is in line with predictions based on a simple rule of mixtures.

Materials with such high elastic modulus and mechanical strength typically fracture in a brittle fashion with no resistance against crack propagation. Instead, the nacre-like architecture of our composites provides toughening mechanisms that progressively increase the resistance of the material against crack propagation (Fig. 4g,i). Force–displacement data obtained for samples with different metal contents indicate that the notched beams exhibit non-catastrophic failure after reaching their maximum load-bearing capacity (Fig. 4d). We characterized the increasing crack-growth resistance of the nacre-like composites by measuring the fracture toughness against crack initiation (*K*_*IC*_) and propagation (*K*_*J*_) as a function of the crack length in single-edge notched beam specimens. We find that the fracture toughness of the nacre-like composite increases by 2 to threefold when a crack propagates for a few hundred micrometers into the structure perpendicular to the orientation of platelets (Fig. 4e). This resistance against crack growth increases the fracture toughness of the composite to up to 15 MPa m^1/2^, which is about 5 times higher than the typical value of pure alumina^39^. The fact that the strength of the composite remains constant in spite of the nearly twofold toughness increase when the iron content changes from 8.9 to 12.4 vol% points out that larger defects might be introduced during processing of the samples with the highest iron concentration. Such defects may result from platelet packing faults in the presence of large iron pockets.

While compositions with 4.0 and 8.9 vol% Fe show a comparable toughening effect, composites with 12.4 vol% display a significantly higher *K*_*IC*_ and *K*_*Jmax*_ values, as well as a steeper increase in fracture toughness as a function of crack length (Fig. 4f). This suggests that the presence of a minimum fraction of iron is crucial to fully benefit from the toughening arising from the metallic phase. The main contribution of the metallic phase to the toughness of the composite is expected to be plastic deformation ahead of the crack tip during the fracture process. SEM images of fractured surfaces provide evidence of plastically-deformed metal between the alumina platelets, indicating that this appears to be indeed an important toughening mechanism in composites containing the highest Fe content (Fig. 4g). In addition to the plastic deformation of the metallic phase, crack deflection by the stiff platelets is another important mechanism that contributes to the enhanced toughness of the nacre-like composites (Fig. 4i).

Microstructural analyses of the fracture surface of nacre-like composites indicate that the alumina platelets are fully wetted by the surrounding metallic phase (Fig. 4h). This contrasts with the poor wetting of the iron particles on the surface of the alumina after complete reduction of the coated platelets at temperatures above 900 °C (Fig. 3b). The formation of hercynite during the sintering of specimens containing 8.9 and 12.4 vol% Fe is probably crucial to enable wetting of the iron phase on the alumina surface and to thus increase the fracture toughness of the nacre-like composites. The absence of such spinel as an interphase between iron and alumina in samples containing 4.0 vol% Fe might in fact be the reason for the lower fracture strength of this composition (Fig. 4b). These observations are in line with a recent study on Ni-containing nacre-like composites^7^. In this case, the formation of a nickel oxide interphase was also found to be essential to enhance the fracture toughness of the metal-ceramic composite.

Compared to previous metal–ceramic composites with nacre-like architecture, our iron-based composites show 30 to 60% higher bending strength (400 MPa) combined with a fracture toughness level nearly as high as that of the toughest Ni-based systems (16 MPa m^1/2^)^7,27^. This set of mechanical properties makes our composites attractive materials for load-bearing structural applications, particularly those where minimum weight is a key requirement (Fig. 4c). Indeed, the metal-ceramic composites developed in this work show 3.2-times higher weight-normalized elastic modulus compared to steels, while keeping the high specific strength of this widely used metal (Fig. 4c).

**Figure 5:**
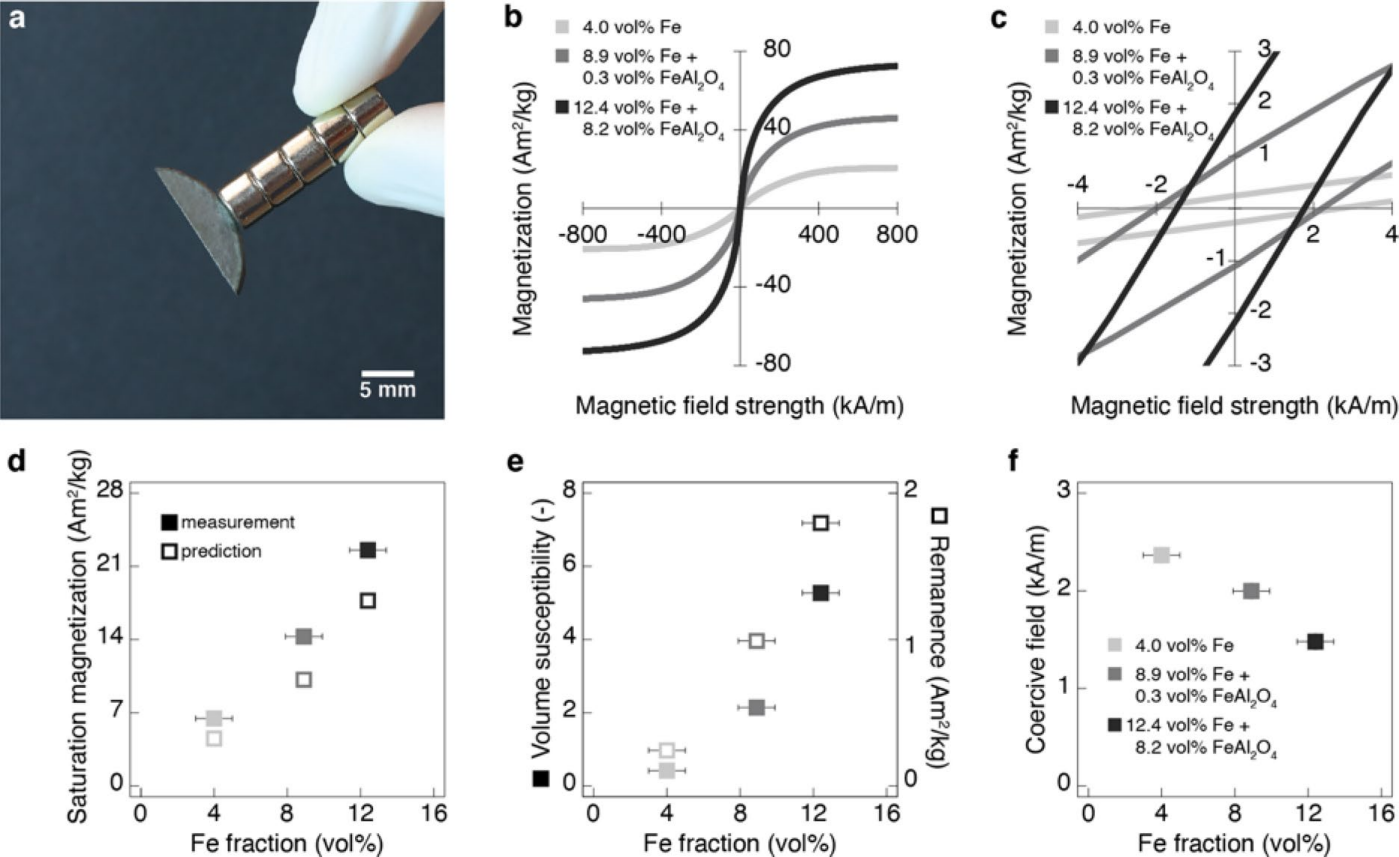
Magnetic properties of the metal-ceramic composites with nacre-like architecture. (**a**) Picture displaying the magnetic attraction between a nacre-like metal-ceramic specimen and a stack of commercial neodymium magnets. (**b**) Magnetization as a function of the applied magnetic field strength of metal-ceramic composites made with increasing volume fractions of iron and hercynite. (**c**) Closer view of the magnetization curves in (**b**) around the origin. (**d**) Saturation magnetization as a function of the volume fraction of metallic iron in the composite. (**e**,**f**) Volume magnetic susceptibility *χ*, the remanent magnetization *M*_*r*_ and the coercive field *H*_*c*_ as function of the content of metallic iron in the specimens.

**Figure 6:**
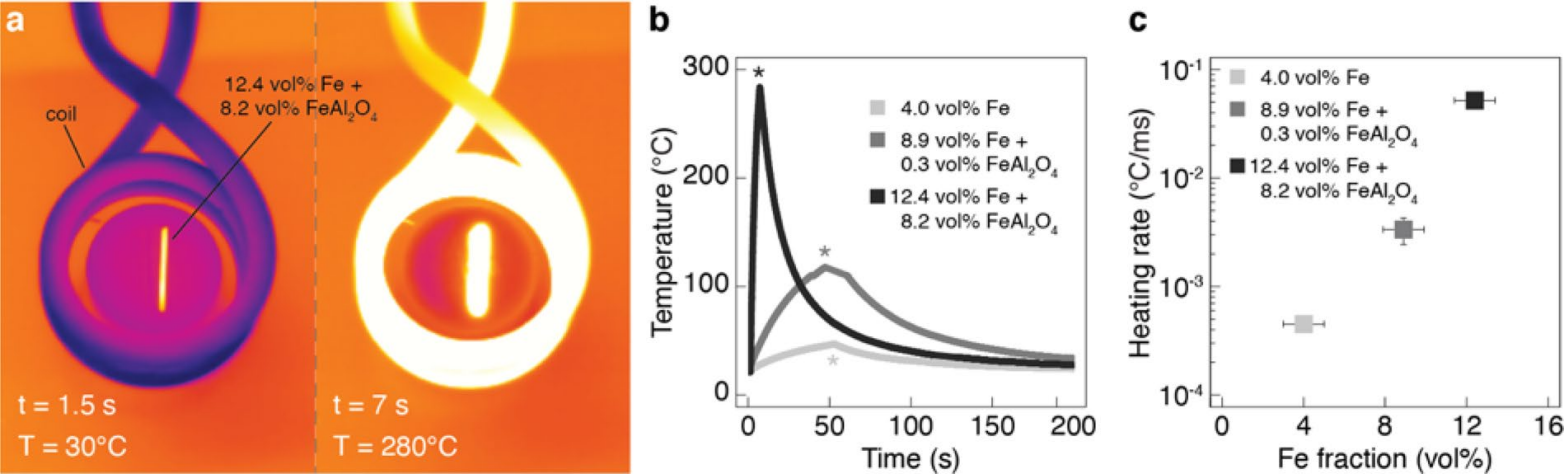
Inductive heating behavior of the metal-ceramic composites upon exposure to oscillating magnetic fields. (**a**) Pictures showing the coil used to generate the magnetic fields and the position of the specimen in the beginning of an experiment (left) and when it reached the peak temperature (right). (**b**) Temperature recorded over time during the inductive heating experiment performed with specimens of different iron fractions. In this experiment, the magnetic field was turned on at time zero and was switched off at the time points indicated with a star symbol inside the plot. (**c**) Average heating rate of composites with varying volume fraction of iron (**b**).

Besides these attractive mechanical properties, the presence of a metallic phase between platelets provides magnetic and electrical functionalities thus far not fully explored in other nacre-like bulk composites. These additional functionalities stem from the electrically-conductive and ferromagnetic properties of the iron phase, which makes the nacre-like composites magnetically-responsive and amenable to inductive heating effects not observed in conventional ceramics (Figs. 5a and 6a). We illustrate such functionalities by measuring the magnetic properties of specimens with varying iron fractions and characterizing their temperature evolution when subjected to induction heating.

The magnetization of ground samples measured as a function of the applied field show that the nacre-like specimens exhibit strong saturation magnetization and relatively low hysteresis losses when subjected to field strengths up to 800 kA/m (Fig. 5b,c). We note that the magnetization obtained at the maximum applied field is taken here as an approximation for the actual saturation magnetization. Because it depends only on the chemical composition of the material^40,41^, the saturation magnetization measured for our composites was compared with the theoretical values expected based on a simple rule of mixture. Such theoretical prediction takes into account the mass fraction and the intrinsic saturation magnetization of the iron and the hercynite present in the composites^42,43^. Comparison of our measurements with the theoretical predictions (Fig. 5d) shows that the saturation magnetizations of our composites lie within the same order of magnitude of the values predicted by the simplified analysis. The stronger discrepancy observed for composites with higher fractions of iron and hercynite might result from the fact that possible amorphous phases contributing to the total magnetic moment are not detected by X-Ray diffraction and thus not considered in the analysis.

In contrast to the saturation magnetization, the shape of the hysteresis loop is strongly dependent not only on the material composition but also on the microstructure, orientation of the ferromagnetic phases, sample shape and domain structure^40,41^. Overall, the relatively small area of the hysteresis loop reveals that the composites show low losses when exposed to oscillatory magnetic fields, which is a typical feature of soft magnetic materials. The shape of the hysteresis loop is determined by the magnetic susceptibility *χ*, the remanent magnetization *M*_*r*_ and the coercivity *H*_*c*_.

To evaluate the effect of the fraction of ferromagnetic phase on these magnetic properties, we plot the values of *χ*, *M*_*r*_ and *H*_*c*_ as a function of the volume fraction of α-iron in the composites. Although it neglects the contribution of the hercynite phase, this simplified analysis should provide insights into possible microstructural features controlling the magnetic properties of the composite. Our results show that the magnetic susceptibility (*χ*) and the remanent magnetization (*B*_*r*_) increase monotonically with the volume fraction of α-iron, whereas the coercive field (*H*_*c*_) decreases with the fraction of this ferromagnetic phase (Fig. 5e,f). The low volume fraction of iron present in the composites leads to volume susceptibility levels that are two orders of magnitude lower than typical values for pure annealed iron^44^. However, the magnetic susceptibility of our composites is high enough to make them responsive to the low magnetic fields applied by household magnets (Fig. 5a). We expect that the magnetic susceptibility of these composites may be further improved in future work through optimization of the microstructure and orientation of the ferromagnetic phase.

Another interesting property arising from the metallic phase is electrical conductivity. The imparted electrical conductivity renders the composite sensitive to oscillating magnetic fields, which can be intentionally used to inductively heat the material in a very short time. Because it combines the low weight and enhanced toughness of nacre-like ceramics with the magnetically-induced heating capability of metals, this functionality cannot be easily achieved using conventional materials.

We demonstrate the inductive heating functionality of our composites by recording the temperature of bulk specimens when subjected to an oscillating magnetic field of amplitude 15 mT and frequency 285 kHz (Fig. 6a). A temperature increase of 255 °C was obtained in as little as 5.5 s using composites containing 12.4 vol% Fe. Measurements on samples with different iron fractions show that the heating rate increases exponentially with the metal fraction (Fig. 6b,c). This strong dependence arises from the fact that a higher metal content increases the amount of heat generated per unit time, the thermal conductivity of the composite and the extent of percolation of the metallic phase. This observation suggests that the investigated iron fractions fall within a range where the metallic phase builds a percolating network throughout the sample volume. Electrical characterization of composites with 4.0, 8.9 and 12.4 vol% Fe shows that the electrical resistance decreases from > 300 MΩ to 119 k$\Omega$ and 2 $\Omega$ as the iron content is increased (Figure S3). In another possible application scenario, the electrically-conductive nature of composites with 12.4 vol% Fe can potentially also be exploited for monitoring crack growth in load-bearing structures, as demonstrated in previous studies^23^.

## Conclusion

The magnetically-assisted assembly and hot pressing of platelets pre-coated with a metallic iron layer is an effective approach for the manufacturing of metal-ceramic bulk composites with nacre-like architecture. With this approach, alumina-based composites with iron fractions up to 12.4 vol% were successfully obtained while maintaining the alumina platelet morphology during the high-temperature reduction and sintering steps involved in the process. Reaction of iron oxides with alumina at temperatures above 600 °C leads to the formation of the spinel interphase hercynite. This interphase is expected to improve the wetting of the metallic phase on the surface of the alumina platelets, thus enhancing the mechanical properties of the resulting composite. Crack deflection at the platelet surface and the presence of a more ductile iron phase in between the alumina platelets lead to a nacre-like architecture that exhibits increasing resistance against crack growth, also known as rising R-curve behavior. As a result of these toughening mechanisms, the bulk composites reach higher mechanical strength and fracture toughness levels comparable to the toughest nacre-like materials reported in the literature. In addition to these remarkable mechanical properties, the metallic iron and hercynite phases also impart soft magnetic response and tunable electrical properties to the final bulk composites. These additional functionalities make the metal-ceramic composites attractive in applications requiring lightweight structural materials that are not only stiff and tough but also sensitive to external magnetic and electrical fields. Such properties are potentially useful for the magnetic shielding of spacecraft and satellites exposed to space radiation. Because it relies on versatile sol–gel chemistry and particle assembly protocols, the proposed manufacturing technology should be applicable to a wide range of metal–ceramic compositions. Future research may identify solid-state reduction routes to increase the iron volume fraction of the nacre-like composites and create metallic alloys with enhanced resistance against crack propagation.

## Methods

### Materials

α-Alumina platelets with a diameter of 7–11 μm and a thickness of 200–250 nm were provided by Merck KGaA (RonaFlair White Sapphire, Darmstadt, DE), whereas iron(III) acetylacetonate 97% (Fe(acac)_3_) and anhydrous benzyl alcohol 99.8% (BnOH) were acquired from Sigma-Aldrich (Buchs, CH). All chemicals were used as received. The alumina platelets used in the fabrication of these composites where chosen due to their size similar to the natural example of nacre and their high intrinsic strength of up to 5 GPa^45^.

### Sol–gel reaction for producing Fe_3_O_4_@Al_2_O_3_ platelets

The as-received alumina platelets were coated with magnetite (Fe_3_O_4_) following the approach proposed by Niebel et al^34^. In a typical sol–gel reaction, 17.5 g of alumina platelets, 17.5 g of Fe(acac)_3_ and 500 mL of BnOH were placed in a 1 L three-neck round-bottom flask. The suspensions were sonicated (ultrasonic homogenizer UP 200s, Hielscher Ultrasonics GmbH, Teltow, DE) for 20 min with a pulse–pause sequence of 1 s–1 s and a relative intensity of 60%. The flask was connected to a condenser with a one-way gas release and sealed with rubber septae attached to a nitrogen gas supply through a needle. To keep an oxygen-free atmosphere, the suspension was bubbled with nitrogen for 30 min and stirred at 600 rpm with a magnetic stirrer heating plate (Hei-End, Heidolph Instruments GmbH Co.KG, Schwabach, DE). Subsequent heating of the suspension to 180 °C in an oil bath for 90 min under a slight nitrogen flow allowed for the sol–gel reaction to take place, which was accompanied by a color change of the mixture from bright red to black. The suspension was then cooled down to room temperature and the resulting magnetite-coated platelets (Fe_3_O_4_@Al_2_O_3_) were vacuum filtered (Filter MN 615 Nr. 01, Macherey-Nagel GmbH Co. KG, Düren, DE). The powder was washed with ethanol and afterwards placed in a drying oven (Universal oven, Memmert GmbH Co. KG, Schwabach, DE) at 60 °C for at least 12 h. The above procedure was carried out using a precursor/platelet mass ratios up to 2. Platelets with thicker magnetite coatings were prepared by performing a second sol–gel reaction on pre-coated platelets (Fe_3_O_4_@ Fe_3_O_4_@Al_2_O_3_).

### Burnout of organic phase from as-coated platelets

The Fe_3_O_4_@Al_2_O_3_ platelets were filled into a ceramic crucible and heated in air to 700 °C in a furnace (P300, Nabertherm, Bremen, DE) using a slow heating rate of 5 °C/min to enable complete oxidation of the organic phase present in the samples. After a dwelling time of 10 min, the powder was cooled down to room temperature and hematite-coated platelets (Fe_2_O_3_@Al_2_O_3_) were obtained.

### Preparation of Fe@Al_2_O_3_ platelets via thermal reduction

In the small-batch study, 0.5 g of Fe_2_O_3_@Al_2_O_3_ platelets were spread on an alumina plate and placed in the quartz tube of a reduction furnace (Gero SR-A 100-500/12, Carbolite Gero GmbH Co. KG, Neuhausen, DE). To ensure an oxygen-free atmosphere, the furnace temperature was first raised to 50 °C and held for 30 min under a constant flow of forming gas (5% hydrogen and 95% nitrogen). The temperature was then raised to the target temperature with a heating rate of 20 °C/min followed by a dwell time of 8 h. Target temperatures between 300 and 1000 °C were chosen for this heat treatment. Afterwards, the oven was turned off and cooled down to room temperature without active cooling.

Similarly to the small-batch study, 10 g of Fe_2_O_3_@Al_2_O_3_ platelets were spread on 3 thin alumina plates placed side by side in the reduction furnace for the up-scaled process. The temperature was raised to 1000 °C at 20 °C/min with a dwell time of 15.5 h. After cooling down, the positions of the samples in the oven were changed and the heating cycle was repeated twice to ensure that all the 3 plates were directly exposed to a stream of forming gas, thus promoting complete reduction of the hematite (Fe_2_O_3_) to pure iron (Fe) on the platelet surface. Fe_2_O_3_@Fe_2_O_3_@Al_2_O_3_ platelets were reduced at 600 °C following the same procedure.

### Magnetically-assisted slip casting for producing green bodies

The Fe@Al_2_O_3_ powder was dispersed in ethanol to form a slurry with a maximum fraction of particles of 20 vol%. To break possible platelet agglomerates, the slurry was sonicated using the same procedure described above for the preparation of platelets for the sol–gel reaction. A green body was then formed via slip casting of the slurry with the aid of a rotating magnetic field. In this magnetically-assisted slip casting (MASC) procedure, the original approach developed by Le Ferrand et al.^6^ was adapted so that the cylindrical graphite die of a spark plasma sintering furnace (SPS) could be directly used as a mold for the green body. With this adaptation, the green body does not need to be transferred from the mold to the die, thus not requiring the use binders in the slurry formulation.

The MASC process was performed following a systematic protocol. Firstly, the inner surface of the die was covered with graphite paper (ProGraphite 0.2, ProGraphite GmbH, Untergriesbach, DE) and the die was placed on top of a gypsum cylinder. The die-gypsum setup was placed close to a neodymium magnet (Death magnet, Webcraft GmbH, Gottmadingen, DE), which was rotated at 100 rpm using an overhead mixer (RE 16, IKA-Werke GmbH CO. KG, Staufen, DE). Secondly, the slurry was poured into the die and the suspended Fe@Al_2_O_3_ platelets were allowed to align under the rotating magnetic field. The gypsum cylinder enabled the removal of the ethanol from the slurry, leading to a solid green body after 2 h. The die with the green body were then placed in a drying oven (Memmert GmbH Co. KG, Schwabach, DE) at 60 °C for 12 h. Finally, the gypsum cylinder was removed and both the top and the bottom surfaces of the dried green body were covered with graphite paper for sintering.

### Spark plasma sintering to produce composites

The consolidation of the green bodies was conducted in a SPS furnace (KCE-FCT HP D 10-SD, FCT Systeme GmbH, Frankenblick, DE). Graphite pistons were inserted in both sides of the 30-mm diameter graphite die already containing the green body and this set was mounted in the SPS furnace between the pressing rams. A pre-force of 3 kN was applied to the pistons and vacuum was pulled in the chamber. Nitrogen was then flushed into the chamber until ambient pressure was reached again. Subsequently, the pressure was raised to 35 kN and then the temperature was increased to 1450 °C within 20 min and the green bodies were sintered at this temperature with a dwelling time of 10 min. The furnace was finally cooled down and the composites were taken out of the graphite die.

### Sample machining

The samples were cut (Accutom-100, Struers GmbH, 8903 Birmensdorf, CH) and ground with a diamond cup wheel to ensure parallel surfaces. For three-point bending tests, both ground surfaces were further polished with abrasive cloth (SiC Foil ranging from P1000 to P4000) and diamond suspensions down to 1 µm (DiaPro, Struers ApS., Ballersrup, DK) before beveling the edges to prevent stress concentration. The samples for SENB tests were pre-notched using a 300-$\upmu$m diameter wire saw (3242, well Diamantdrahtsägen GmbH, Mannheim, DE). The notches were manually sharpened using a razor blade (35010, MARTOR KG, Solingen, DE) and 250 nm diamond paste (DP-Paste P, Struers ApS., Ballersrup, DK) to yield notch radii lower than 25 μm. The dimensions of the notches were estimated with a digital microscope (VHX-6000, Keyence Deutschland GmbH, Neu-Isenburg, DE). The final sample sizes varied depending on the quantity of coated platelets used in the initial suspension. The smallest sample sizes (length l x width b x thickness w) were 15 × 1.2 × 1.2 mm^3^ and 15 × 0.6 × 1.2 mm^3^ for the three point bending and SENB tests, respectively. The largest three-point bending and SENB samples were, respectively, 15 × 2.4 × 2.4 mm^3^ and 15 × 1 × 2 mm^3^ in size. Size effects are not expected to play a significant role for samples with these dimensions^6^.

### Microscopic characterization

High-resolution micrographs of platelets and sample fracture surfaces were taken with the in-lens detector of a field-emission scanning electron microscope (Leo Gemini 1530, Carl Zeiss AG, Oberkochen, DE). Prior to imaging, specimens were sputtered with a 3 nm platinum coating in a sputter coater (CCU-010, safematic GmbH, Bad Ragaz, CH) under argon atmosphere. Energy-dispersive X-Ray spectroscopy (UltraDry II, Thermo Fisher Scientific GmbH, Dreieich, DE) was used to determine the local concentrations of iron and oxygen atoms along specific directions of the sample. Optical analysis of the samples was conducted with a digital microscope (VHX-6000, Keyence Deutschland GmbH, Neu-Isenburg, DE).

### Analysis of crystallographic phases

The identification and quantification of crystallographic phases in the synthesized powders and sintered samples were conducted with an X-Ray diffractometer (XPert Pro MPD, Malvern Panalytical B.V., Almelo, NL) using a Bragg–Brentano geometry and Cu*-K*$\alpha$_*1*_ radiation, operated at 40 kV and 45 mA. To achieve high precision and verify that the equilibrium condition has been reached in high temperature experiments, each single measurement consisted of five consecutive scans with the 2$\theta$ angle ranging from 5° to 120° in 0.034° steps. Time intervals of 270 s were used between steps, resulting in scan time of around 10h20. The phases were quantified via Rietveld refinement of the diffractograms performed with the software HighScore Plus (4.7a, Malvern Panalytical B.V., Almelo, NL). The uncertainties associated with the crystalline phase fractions obtained through Rietveld analysis were estimated to be under 1 vol% based on the refinement quality. All of the observed Bragg peaks could be associated with one of the reported phases. Amorphous phases were not considered during phase quantification.

The strong absorption of Cu radiation by the ferrous shell around the alumina platelets may lead to shielding effects and thus to an underestimation of the fraction of alumina phase. Based on SEM investigations, we estimate the thickness of the Fe-containing envelope to be at maximum 500 nm, which would lead to X-ray absorption by the coating of roughly 10%. Since bulk- and micro-absorption within the Fe-containing crystallites partly compensate for this effect, a complex correction of the raw data was omitted. After sintering, Fe does no longer encloses the platelets completely, but aggregates in larger grains (Fig. 4h). Therefore, micro-absorption of the ferrous phases might even dominate, leading to underestimated ferrous phases. In any case, absorption effects mainly affect the quantification of alumina versus Fe-containing phases. The relative fraction of the ferrous phases is significantly less influenced by absorption, since their absorption coefficients are more similar.

### Mechanical characterization

Flexural and single-edge notched bending (SENB) tests were carried out as specified in the ASTM designations C 1161–18 and C 1421–18, respectively^36,37^. Measurements were performed in an universal mechanical testing machine (Shimadzu AGS-X, Shimadzu Schweiz GmbH, Reinach BL, CH) using a three-point bending setup with a loading speed of 1 μm/s and a span of 20 mm for flexural tests and 12 mm for SENB tests. The size of the main crack propagating from the notch was determined from changes in the compliance of the specimens during the SENB tests^46^. The relation $C=u/F$ was used to calculate the compliance $C$ for each data point, where $u$ and $F$ are the total displacement and the measured force, respectively. The crack length $a$ was calculated for the data points acquired after crack initiation, which is indicated by a change in the compliance of the sample. Taking the notch size as initial crack length ${a}_{1}$, the actual crack size ${a}_{i}$ was recursively calculated from ${a}_{i-1}$ using the expression:

1 $${a}_{i}={a}_{i-1}+\left(\frac{W-{a}_{i-1}}{2}\right)\left(\frac{{C}_{i}-{C}_{i-1}}{{C}_{i}}\right),$$

where $W$ is the specimen thickness and ${C}_{i}$ is the compliance value calculated in the $i$th step.

The values of crack length were used to calculate the energy dissipated during stable crack propagation using the J-integral approach^47^. The total energy applied to the sample consists of an elastic and a plastic component. The elastic component ${J}^{el}$ was obtained for each step from the linear-elastic mechanics relation ${J}^{el}={K}_{I}^{2}/{E}^{^{\prime}}$, where ${K}_{I}$ is the stress intensity factor and ${E}^{^{\prime}}=E/\left(1-{\upsilon }^{2}\right)$ is the elastic modulus of the specimen in plane strain conditions. The plastic component ${J}_{i}^{pl}$ was calculated with the following expressions:

2 $${J}_{i}^{pl}=\left\{\begin{array}{ll}\frac{1.9{A}_{i}^{pl}}{B{b}_{i}},& i=1\\ \left[{J}_{i-1}^{pl}+\left(\frac{1.9}{{b}_{i-1}}\right)\left(\frac{{A}_{i}^{pl}-{A}_{i-1}^{pl}}{B}\right)\right]\left[1-\frac{{a}_{i}-{a}_{i-1}}{{b}_{i-1}}\right],& i>1\end{array}\right.$$

where ${A}_{i}^{pl}$ is the plastic area under the load–displacement curve, $B$ is the specimen lateral dimension and ${b}_{i}$ is the length of the uncracked ligament at the $i$th step.

The stress intensity factor ${K}_{J,i}$ taking both the elastic and the plastic contributions into account was then calculated for every crack length ${a}_{i}$ using the following relation, as specified in ASTM designation E 1820–01^38^:

3 $${K}_{J, i}=\sqrt{\left({J}_{i}^{el}+{J}_{i}^{pl}\right){E}^{^{\prime}}}.$$

### Magnetic characterization

Magnetic hysteresis curves were measured on a vibrating sample magnetometer at the Laboratory of Natural Magnetism, ETH Zurich (MicroMag 3900, Princeton Measurements Corporation, Princeton, USA) by applying magnetic fields up to 800 kA/m. The slope of the hysteresis curves at the origin was used as an estimate of the magnetic susceptibility, whereas the induced magnetization at the maximum applied field was taken as an approximation for the saturation magnetization.

### Inductive heating experiments

Heating measurements were performed by subjecting the composite samples to an alternating magnetic field with a frequency of 285 kHz and an amplitude of 15 mT generated by an induction coil (EasyHeat, Ambrell Corporation, Cheltenham, UK). The samples were placed inside the copper coil of the heater and the temperature on the specimen surface was recorded as a function of time using an infrared camera (Testo 885–2, 33 Hz, Testo SE & Co. KGaA, Lenzkirch, DE). The copper coil had three turns and a diameter of 50 mm.

## Supplementary Information

Supplementary Figures.
