# Supplementary material for: Tough metal-ceramic composites with multifunctional nacre-like architecture
Source: Sci Rep. 2021 Jan 15;11:1621. doi: 10.1038/s41598-021-81068-z (PMC7810751; doi:10.1038/s41598-021-81068-z)
Supplement: Supplementary file 1 — Supplementary Figures. [file 41598_2021_81068_MOESM1_ESM.docx]

Supporting Information

Tough metal-ceramic composites with multifunctional nacre-like architecture

Erik Poloni^1^, Florian Bouville^1+*^, Christopher H. Dreimol^1^, Tobias Niebel^1^, Thomas Weber^2^, Andrea R. Biedermann^3^, Ann M. Hirt^4^, André R. Studart^1*^

^1^Complex Materials, Department of Materials, ETH Zürich, 8093 Zürich, Switzerland
E-mail: [f.bouville@imperial.ac.uk](mailto:f.bouville@imperial.ac.uk), [andre.studart@mat.ethz.ch](mailto:andre.studart@mat.ethz.ch)

^2^X-Ray Service Platform, Department of Materials, ETH Zürich, 8093 Zürich, Switzerland

^3^Institute of Geological Sciences, University of Bern, 3012 Bern, Switzerland

^4^Institute of Geophysics, ETH Zürich, 8092 Zürich, Switzerland

^+^ now at: Centre for Advanced Structural Ceramics, Department of Materials, Imperial College London, South Kensington Campus, SW7 2AZ, United Kingdom


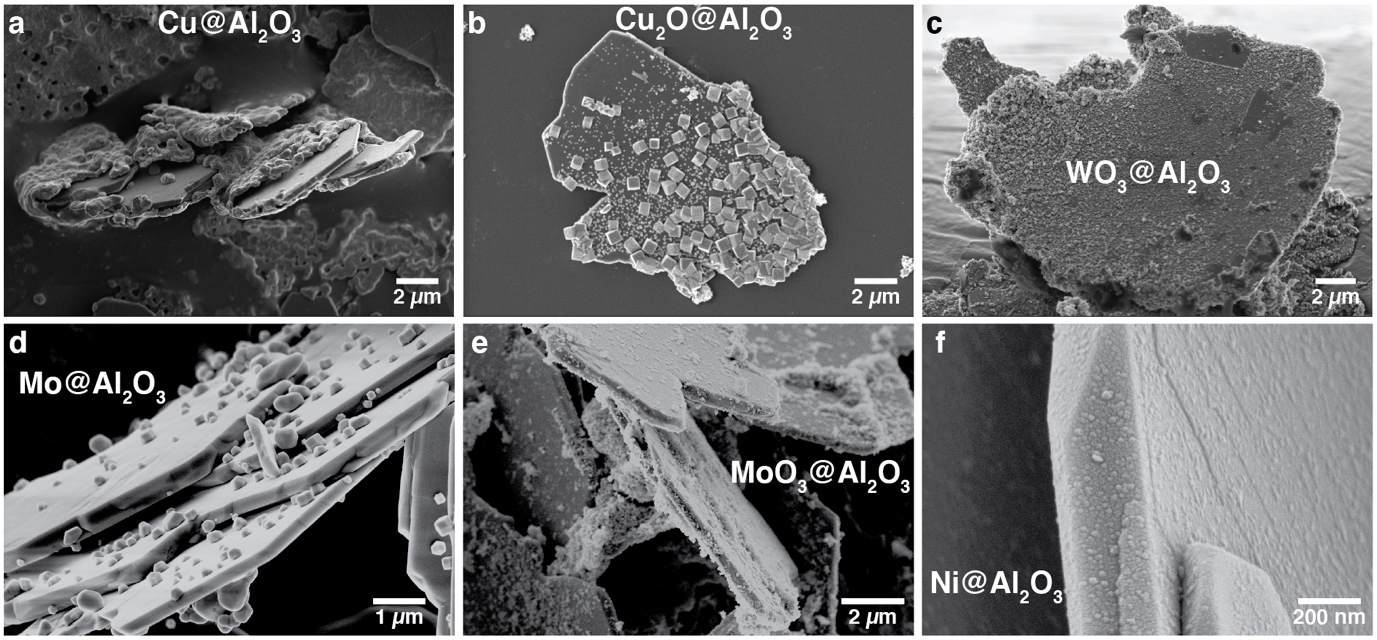


**Figure S1**: Alumina platelets coated with layers of different chemistries using the proposed sol–gel reaction. The SEM images show (a) copper, (b) copper oxide, (c) tungsten oxide, (d) molybdenum, (e) molybdenum oxide and (f) nickel coatings on alumina platelets.

**
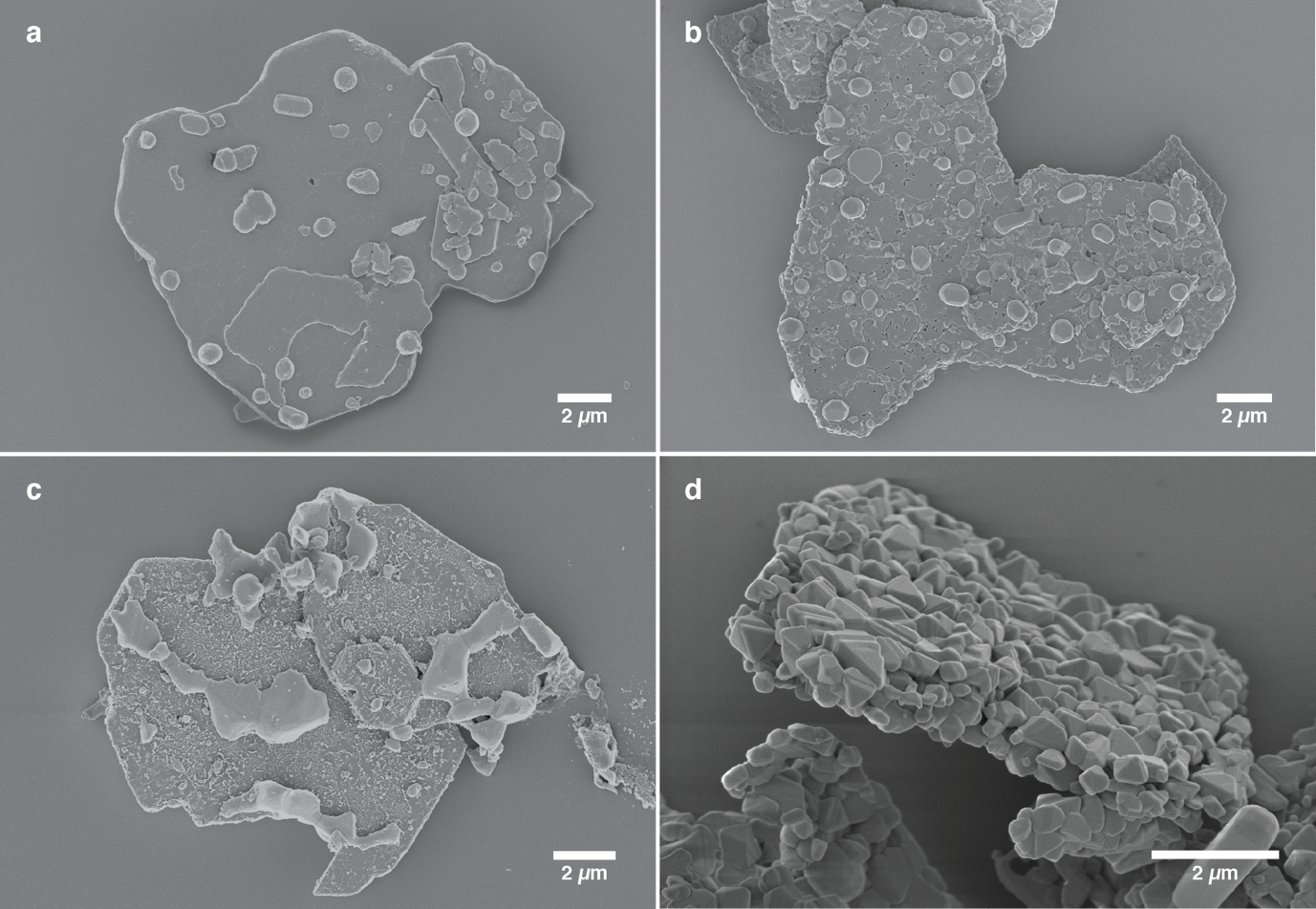
**

**Figure S2**: Morphology after 15-h reduction of alumina platelets coated with different fractions of hematite at different temperatures: (a) 10 vol% of hematite reduced at 1000°C, (b) 23 vol% at 1000°C, (c) 43 vol% at 600°C and (d) 43 vol% at 1000°C. The SEM image of the system containing 43 vol% hematite reduced at 1000°C shows that the morphology of the original platelets (Figure 2b-d) changes significantly under such thermal treatment, justifying the need of a lower reduction temperature.


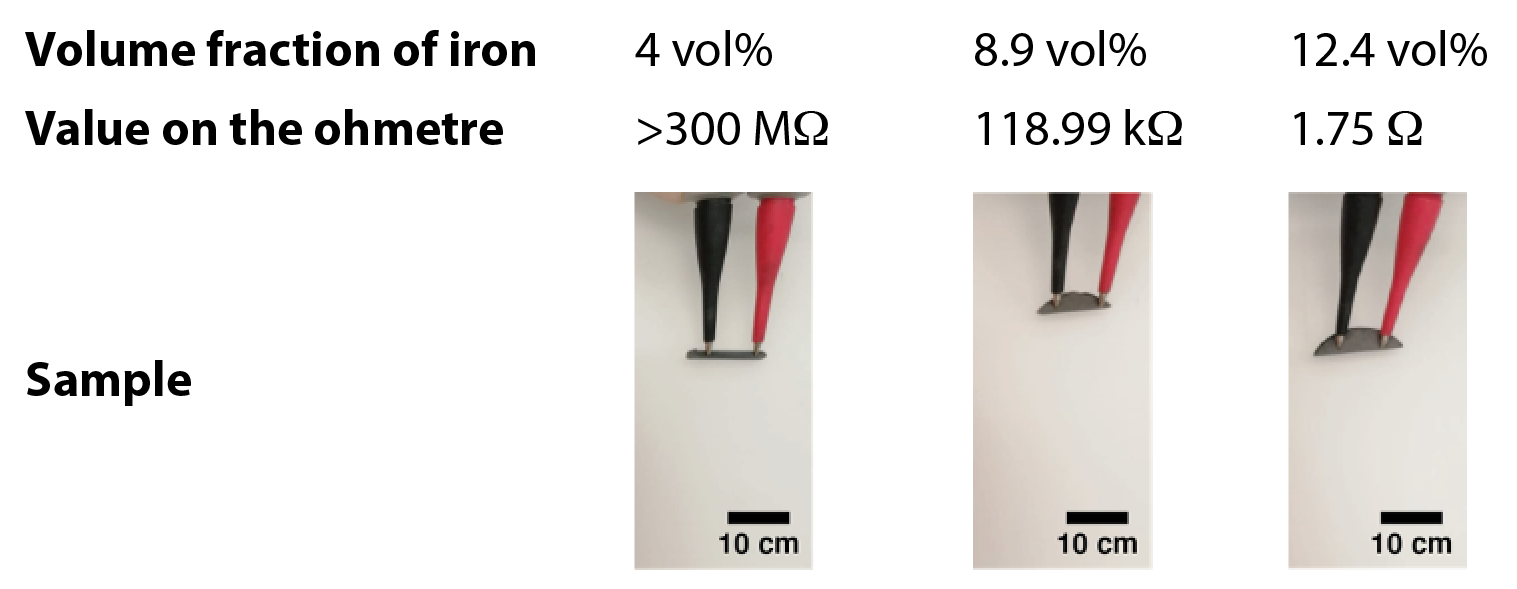


**Figure S3**: Electrical resistance of composites with (a) 4.0, (b) 8.9 and (c) 12.4 vol% Fe.
